# Declarative Learning-Based Programming as an Interface to AI Systems

**DOI:** 10.3389/frai.2022.755361

**Published:** 2022-03-14

**Authors:** Parisa Kordjamshidi, Dan Roth, Kristian Kersting

**Affiliations:** ^1^Department of Computer Science and Engineering, Michigan State University, East Lansing, MI, United States; ^2^Department of Computer and Information Science, University of Pennsylvania, Philadelphia, PA, United States; ^3^Department of Computer Science, Centre for Cognitive Science, TU Darmstadt, Darmstadt, Germany

**Keywords:** machine learning, artificial intelligence, integration paradigms, programming languages for machine learning, declarative programming, probabilistic programming

## Abstract

Data-driven approaches are becoming increasingly common as problem-solving tools in many areas of science and technology. In most cases, machine learning models are the key component of these solutions. Often, a solution involves multiple learning models, along with significant levels of reasoning with the models' output and input. However, the current tools are cumbersome not only for domain experts who are not fluent in machine learning but also for machine learning experts who evaluate new algorithms and models on real-world data and develop AI systems. We review key efforts made by various AI communities in providing languages for high-level abstractions over learning and reasoning techniques needed for designing complex AI systems. We classify the existing frameworks based on the type of techniques and their data and knowledge representations, compare the ways the current tools address the challenges of programming real-world applications and highlight some shortcomings and future directions. Our comparison is only qualitative and not experimental since the performance of the systems is not a factor in our study.

## 1. Introduction

The goal of conventional programming is to automate tasks that are explainable as a set of step-by-step instructions. The main goal of AI has been to develop programs that make intelligent decisions and solve real-world problems, possibly dealing with “messy" real world input that could make it difficult to handle using “conventional" programming. The earlier AI problem solvers were expert systems that attempted to model the way experts reason and make decisions using a set of logical rules. Programming languages like Lisp^1^ and Prolog were designed to make programming such systems easy even for non-expert users. The idea was to represent the domain knowledge using a set of logical rules, and use the rules in a logical reasoning process hidden from the programmers.

From the traditional AI perspective, this is a declarative programming paradigm where we program for the *what* and not the *how*. The expert programs could go beyond an independent set of rules and turn to logical programs with a Turing-complete expressivity, supporting logical inference, for example, by unification and resolution. However, real-world problems are complex and often involve many interdependent components. Most importantly, there is a need to interact with naturally occurring data — text, speech, images and video, streams of financial data, biological sequences—and to reason with respect to complex concepts that often cannot be written explicitly in terms of the raw observed data. It has become evident that formalizing complex problem solving using programming a finite set of deterministic logic-based rules is not possible, nor is it possible to write a conventional structured program, even with a Turing-complete language, for supporting intelligent decision-making based on naturally occurring data. Consequently, there has been a rapid paradigm shift from formal modeling to data-driven problem solving. This has affected not only core AI problems like natural language understanding, computer vision, and game playing but also real-world problems in many areas including cognitive sciences, biology, finance, physics, and the social sciences. It is becoming progressively common for scientists to think about data-driven solutions using machine learning techniques.

Machine learning has been defined as the study of computer programs that can learn to perform tasks from experience/data (Mitchell, 1997). However, this is not the currently dominating view of machine learning. In the current view, programs are reduced to functions of predefined form that map input to output and learning is an optimization process driven by an objective function also of a predefined form. Thus, machine learning focuses on learning models based on classification, regression, or clustering objective functions rather than generic programs and problem solvers for arbitrary tasks. Nevertheless, considering machine learning models as “computer programs” provides a larger capacity to express and explain models that can solve complex real-world problems using various learning and reasoning components. Therefore, we suggest that this perspective needs to be systematically investigated.

Current machine learning (ML) and AI technologies do not provide easy ways for domain experts who are not ML/AI experts to develop applications; as we show later, they provide rather cumbersome solutions along multiple dimensions. Even for AI experts when inventing new techniques, they need to evaluate those on messy real-world data rather than on well-formed toy problems, which means that both users and developers will need to spend a tremendous amount of time and effort due to missing values, formatting errors, anomalies, not to mention “simply” the ambiguity and variability inherent in naturally occurring data.

Building today's complex AI systems, however, requires extensive programming time and skills in addition to the ability to work with various reasoning and learning paradigms and techniques at a rather low level of abstraction. It also requires extensive experimental exploration for model selection, feature selection, and parameter tuning due to lack of theoretical understanding or tools that could be used to abstract over these subtleties. Conventional programming languages and software engineering paradigms have not been designed to support the challenges faced by users of AI Systems. In particular, they were not designed to deal with messy, real-world data at an appropriate level of abstraction. While the use of data-driven methods, incorporating expert, domain and task specific information, is always important at the application level, programming expert knowledge into current data-driven models in an intuitive way is highly challenging. There is a need for innovative paradigms that seamlessly support embedded trainable models, abstract away most low-level details, and facilitate reasoning with respect to these at the right level of abstraction.

We believe that this problem is at the heart of many interesting and fundamental research questions and that it goes beyond simply developing good toolboxes and libraries for ML and AI approaches based on existing techniques. Particularly, it requires integrating well-established techniques; dealing with multiple research challenges in data and knowledge representation and reasoning; integration of various machine learning formalisms; and innovations in programming languages and software development.

To help closing this gap and facilitate progress in developing what we call here *Systems AI*, we survey key efforts made in this direction. We emphasize the need to use some fundamental declarative ideas such as first-order query languages, knowledge representation and reasoning techniques, programming languages for multi agent systems, database management systems (DBMS), and deductive databases (DDB). We need to place these ideas within and around ML formalisms including classical ML tools, deep learning libraries and automatic differentiation tools, and integrate them with innovative programming languages and software development techniques, as a way to address complex real-world problems that require both learning and reasoning models.

We proceed as follows. In the rest of Section 1, we categorize the requirements of the AI-application programming, then we review the main exiting paradigms and their key characteristics. In Section 2, we clarify the ways existing paradigms address these requirements. In Section 3, to conclude our review, we advocate for the need for an integrated paradigm. We then clarify the type of abstractions needed to address the shortcomings of the existing paradigms. We use the term (declarative) Learning based programming, coined by Roth (2005), to refer to the ideal language that interfaces and helps designing complex learning-based AI systems and follows a declarative style.

### 1.1. AI Application Requirements

**Figure 1:**
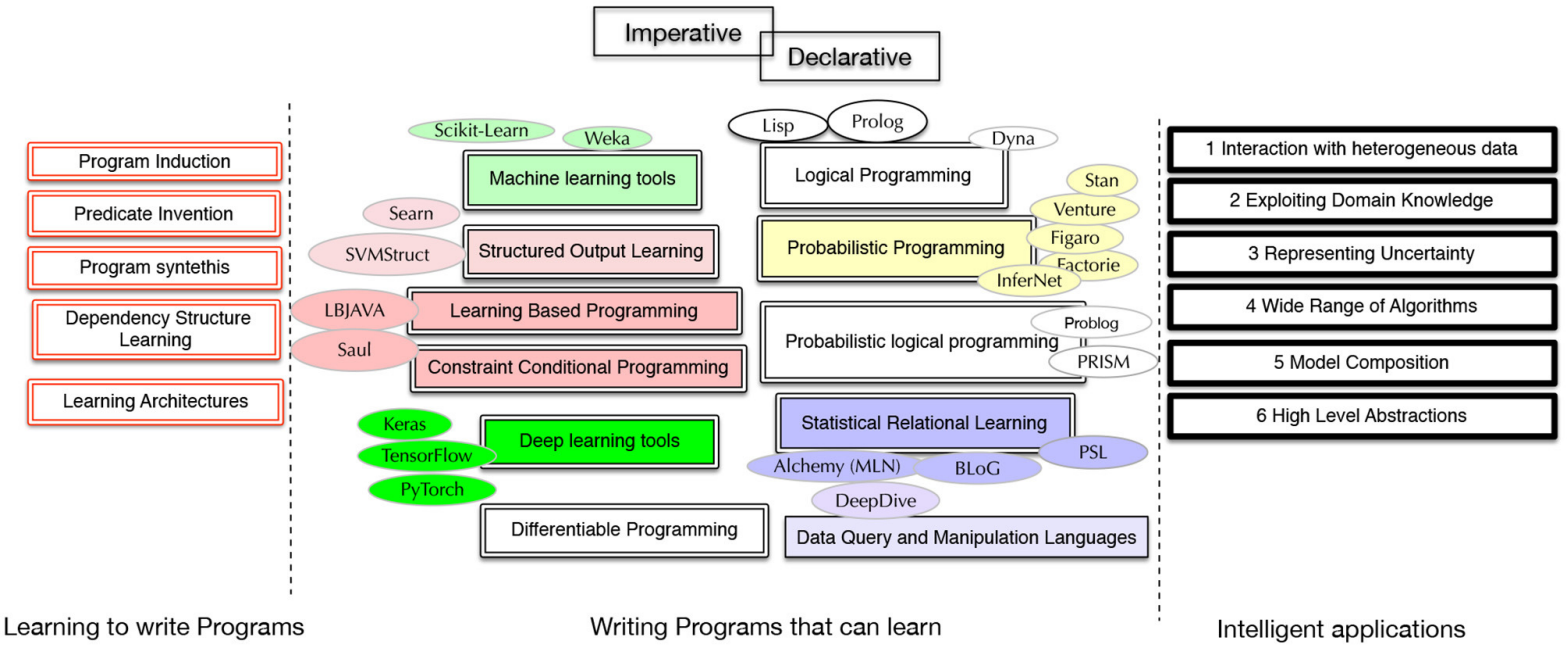
Related paradigms and example frameworks.

We identify the following criteria as areas of need to enrich existing frameworks with capabilities for learning-based programming (Roth, 2005) and for designing complex AI applications and systems. We also point to a number of questions related to the characteristics of programming languages that enable those requirements. See Figure 1 for a summary.

1. Easy interaction with raw, heterogeneous data: The key question is *how is communication with the data performed in the exiting frameworks?*
2. Intuitive means to specify and use domain knowledge: *What kind of knowledge is needed? Should it be declarative or imperative? How should it be specified?*
3. Express uncertainty in data and knowledge: *How should uncertainty be represented? Which underlying formalisms can be used? What kind of expressive power is needed?*
4. Access to various learning, inference and reasoning techniques: *What underlying algorithms are to be supported?*
5. Ability to reuse, combine and chain models and perform flexible inference with complex models/pipelines: *How can we support building end-to-end models from heterogeneous components?*
6. High-level and intuitive abstractions for specifying the requirements of applications: *What should be expressed in a learning-based program? The training objective function? The data? The knowledge? Do we need programs that can learn, or do we need conventional programming that includes learning-based components? Should it be a language or a library? What should be the level of automation? Can we learn the programs automatically?*

To discuss the existing related paradigms and the key techniques addressing them, we will use a running example—designing an intelligent model solving a simple entity-mention-relation (EMR) extraction task—and assume populating a knowledge graph using such information:

**Given** an input text such as “*Washington works for Associated Press*., ” **find** a model that is able to extract the semantic entity types (e.g., people, organizations, and locations) as well as relations between them (e.g., works for, lives in), and generate the following output: *[Washington]_person_ [works for]_worksFor_ [Associated Press]_organization_*. The chunks of text, along with the labels, will populate a knowledge graph that contains nodes that correspond to entities, and edges that correspond to relations between them. Note that by “population” we mean that nodes and edges are added for entities and relations, and strings are assigned to these as attributes, identifying the entity type or relation, respectively.

### 1.2. Related Existing Paradigms

The AI community has developed various proposals to address the aforementioned requirements for designing intelligent applications. Indeed, there have been various proposals within the AI community that address the aforementioned requirements for designing intelligent applications. We will first review the related communities and some of the frameworks to provide the big picture. We will refer back to these frameworks in the following sections when we compare them. The key issues with these frameworks are that:

- One still needs deep mastery of ML and AI techniques and methodologies in order to engineer AI systems, and this knowledge far exceeds what most application programmers have.
- None of these paradigms covers all the requirements in one unified framework.

Figure 1 shows a rough picture of various paradigms that are related to learning-based programming in one way or another. The right side shows the six requirements from intelligent applications. In the middle, we point to eight different paradigms, some tightly related, that deal with languages and tools for high-level machine learning and declarative programming. The left side shows concepts related to learning to learn programs.

**Probabilistic Programming Languages (PPLs)**. These languages are designed to describe probabilistic models and perform probabilistic inference. Given that estimating the parameters of probabilistic models and making predictions based on probabilistic inference is one of the main class of techniques used in machine learning, probabilistic programming languages help users to design and use probabilistic models without worrying about the underlying training and inference algorithms. Examples include Figaro (Pfeffer, 2016), Venture (Mansinghka et al., 2014), Stan (Carpenter et al., 2017), and InferNet (Minka et al., 2014).

**Probabilistic Logic Programming (PLP)**. The aim of these languages is to combine the capacities of probability theory and deductive logic. When compared to probabilistic programming languages, in addition to the logical reasoning aspect, they bring in capabilities of higher order and compact logical representations of the domain knowledge. The parameters of the PL programs are trained from data, and they can make predictions based on probabilistic logical reasoning. Examples are ProbLog (De Raedt et al., 2007) and PRISM (Sato and Kameya, 1997).

**Statistical Relational Learning (SRL)**. This discipline deals with languages that are able to describe complex relational structures and express uncertainty. They do not always rely heavily on logical reasoning but usually exploit a subset of first order logic to express structures. The structures are used during training machine learning models and making inference under uncertainty. Examples are Markov logic networks (Richardson and Domingos, 2006), Probabilistic soft logic (Broecheler et al., 2010) and BLOG (Bayesian Logic) (Milch et al., 2005). The relational and logical representations bring in the capabilities of more compact representations, parameter tying and efficient lifted inference (De Salvo Braz et al., 2005) in SRL models as well as in probabilistic logical models. With a different perspective from these examples, Constrained Conditional Models use the relational representations in learning in the form of logical constraints (Roth and Yih, 2004; Chang et al., 2012).

**Neuro-symbolic learning**. Neuro-Symbolic languages (d'Avila Garcez et al., 2009) aim at integrating deep neural learning and symbolic reasoning. Knowledge is typically represented in symbolic form, whereas learning and reasoning are performed by a neural network that is usually a differentiable program. There are many recent techniques and algorithms proposed for combining neural and symbolic paradigms (Hu et al., 2016; Wang and Poon, 2018; Xu et al., 2018; Dong et al., 2019; Nandwani et al., 2019), however not many generic libraries are available. The recent extensions of Problog that is Deep Problog (Manhaeve et al., 2018) and a similar framework based on interfacing Datalog and deeplearning libraries, that is, Scallope (Huang et al., 2021) are examples of this direction of research. Problog is based on prolog's logical formalism and Scallop follows a similar approach on the basis of Datalog. There are new emerging libraries built on top of current deep learning tools that provide the possibility of integration of logical and symbolic constraints into neural models (Faghihi et al., 2021; Ahmed et al., 2022).

**Agent-oriented Programming Languages (AOP)**. These languages operate on high-level semantic abstractions to design and communicate with intelligent agents that interact with an environment and make decisions (Georgeff and Lansky, 1987; Shoham, 1993). In particular the models are developed based on high-level primitives such as goals, beliefs, desires, plans, actions and cognitive elements in intelligent agents. The abstractions are beyond low-level algorithms and mathematical tools and help the integration of procedural and declarative knowledge in decision making by AI systems. A more recent work (Belle and Levesque, 2015), proposes a belief-based programming language for stochastic domains that bridges the classical agent-based programming with probabilistic programming to address uncertainty and noise.

**Learning-Based Programming**. The main idea is to look at learning models as first class objects that are able to extract features and make uncertain decisions. It focuses on the ways that these first class objects can be composed and constrained to form global models to predict complex structures (Roth, 2005). The LBJava language (Rizzolo and Roth, 2010; Rizzolo, 2011a) and Saul library (Kordjamshidi et al., 2015) are based on this perspective. DomiKnowS (Faghihi et al., 2021) is a very recent declarative framework that pursues a similar idea.

**Classical Machine Learning Toolboxes**. These are usually libraries designed in general purpose languages and call the training and prediction based on classical classifiers and regressors. These cover broad ranges of classification, clustering and regression algorithms that are applied on a form of flat vector representations. Examples are Python Scikit-learn libraries^2^ and WEKA (Witten et al., 1999), among others.

**Structured Learning Tools**. These tools go beyond classical machine learning toolboxes by allowing the programmer to encode the structure of the multiple output variables and perform inference during training. SVM-struct^3^, JLIS^4^ and SEARN (Daumé et al., 2014) are examples.

**Deep Learning Tools and Languages**. These are usually libraries within general purpose languages and help with designing deep learning architectures. Examples are PyTorch^5^ and TensorFlow (Abadi et al., 2016) among others.

**Differentiable Programming**. This is a recent paradigm that is used as the basis of deep learning tools. Imperative programs can be written in terms of a sequence of computations that include differentiable operations where differentiations are calculated automatically (Baydin et al., 2017). In the deep learning case, the program's parameters are optimized by back propagation of the errors based on the automatically derived gradients of an error function given data to train the models and produce correct outputs given the inputs.

**Data Query and Manipulation Languages**. Since learning is data-driven, a language for accessing and querying data in both input and output sides is an essential part of a learning-based program in many applications. The ideas in deductive databases (Bárány et al., 2017) are relevant as they provide platforms for integration of data and first-order knowledge for inference. The probabilistic databases are also highly related because of their capacity to handle uncertainty in answering database queries and making probabilistic inference (Suciu et al., 2011; den Broeck and Suciu, 2017).

We refer back to Figure 1 when we discuss the existing work in the next section. We connect the notion of *writing* learning-based programs to that of *learning* learning-based programs, which, in turn, is related to program synthesis, program induction and learning end-to-end differentiable programs. Our goal is to organize the various lines of work related to developing languages for designing machine learning applications and highlight some fundamental research questions that can open new avenues for research on machine learning, programming and developing AI systems.

## 2. How do Existing Paradigms Address Application Requirements?

Given the aforementioned requirements and the key questions to be addressed, in this section, we explore their relationship with existing frameworks. This allows us to discuss the shortcomings of the existing frameworks. We use the EMR example to clarify the concepts when needed.

### 2.1. Interaction With Heterogeneous Data

For real-world applications, organizing and using data is an essential starting point for learning-based programs. For example, in the EMR task, we interact with raw text data (strings). We need to extract useful abstractions from the text and put raw text into a structure such as a relational database, a parsing tree or any other structured representation for easy access and use in other tasks. We may also want to associate properties of text chunks with them; these could be their semantic types or even a continuous representation (embeddings). In this section, we point to some of the existing frameworks that facilitate such interactions with both structured and unstructured data in various forms.

**Unstructured Data**. Many real-world systems need to operate on heterogeneous and unstructured data such as text and images. To structure the raw and sensory data, we need information-extraction modules that could be complex intelligent components themselves. In the EMR task, an initial required step, before any semantics to be inferred via learning components, is chunking. Chunking is splitting the sentence^6^ into a number of phrases such as [Washington][Works For][Associated Press][.]. This is a challenging learning task on its own but also provides a primary structure that classifiers can operate on. Such complex prepossessing steps can also be learned jointly with the main target tasks.

Some older research tried to combine information extraction modules with relational DB systems and use standard query languages for retrieving information such as SystemT (Krishnamurthy et al., 2009). Different systems were designed for processing textual data and provide a regular expression interface language to query linguistic features directly from text (Broda et al., 2013). To facilitate working on unstructured data, systematic efforts have been made to design unified data structures for processing textual data and tools that can operate on those data structures. A well-known example of such a universal data structure is Unstructured Information Management Architecture (UIMA) (Ferrucci and Lally, 2004) that can be augmented with natural language processing (NLP) tools that provide lexical, syntactic and semantic features (Sammons et al., 2016). UIMA focuses on providing a specific internal representation of raw data (it covers text and also extended to multiple modalities). However, this established infrastructure does not support declaring a data model with an arbitrary structure. In other words, it is designed for text with a fixed linguistic data model for documents, sentences and other linguistic units; it does not allow defining arbitrary concepts and defining their relationships based on the problem. The above mentioned information extraction system, SystemT, is equipped with very well designed and efficient query languages based on their fixed internal data model (Krishnamurthy et al., 2009).

While there has been several such efforts to process unstructured data, there is a disconnection between such systems and machine learning tools. On one hand, such systems do not address learning and inference, that is, their functionality is independent from the design of learning models. However, they could be used as *sensors*^7^ for information extraction in designing learning models. On the other hand, existing machine learning tools do not address the issues involved in working with unstructured data. Current ML tools such as WEKA (Witten et al., 1999) and newer Python and deep learning packages (Abadi et al., 2016) provide easy access to learning algorithms but typically only support feature vectors/tensors in a specific format, making it difficult to work with raw or structured data. This is the obvious gap in the existing systems for applying machine learning on raw data.

**Relational and Graph Data**. Many applications require dealing with complex and structured data. Organizing, manipulating and efficient querying from data has been addressed by relational database management systems based on relational representations and standard query languages. These systems traditionally do not accommodate learning-based models nor support the design of end-to-end learning models using raw data to extract information and put them into a queryable structure as needed for example in EMR task. For EMR, however, we want to learn to extract the entities and relationship and put them into a database for efficient and easy use.

Providing ML capabilities on top of relational databases has been persuaded, for example, in the DeepDive system (Zhang et al., 2017) and the follow up work Snorkle (Ratner et al., 2017), where first order logical SQL expressions form a Markov logic network. However, such a connection can be done to any relational probabilistic model (De Raedt et al., 2016). These logical expressions are grounded for parameter estimation and for inference and predictions over relational data. In the relational logic-based learning framework of kLog (Frasconi et al., 2014), one can use black box classifiers based on relational features which are represented using a logical style and implementations of Datalog. Relational data and relational features can be queried and used directly in machine learning models. The possibility of programming for the objective functions by SQL in DBMS environment and forming learning objectives was followed in the LogicBlox (Aref et al., 2015) and RELOOP (Kersting et al., 2017) systems. Another example of the relational learning paradigm is the Saul (Kordjamshidi et al., 2015) language, which is equipped with in-memory graph queries that can be directly used in learning models as features or for constructing learning examples. Moreover, the queries can form global structural patterns between inputs and outputs (Kordjamshidi et al., 2016, 2017).

One shortcoming of these frameworks that integrate structured and relational data into learning is that they cover only a specific learning approach and do not provide the flexibility of working with various learning and inference algorithms. Moreover, they offer no flexibility in feature design when working with raw data; in other words, the initial graph/structure should be encoded in a specific way and given to the model.

**Feature Extraction**. One central goal of interaction with data in learning-based programs is to facilitate defining and extracting features from various data sources for learning models. Typically, feature engineering includes (a) the ability to obtain low-level sensory properties of learning examples (*e.g*., the length of a phrase or the lemma of its words); (b) the capability of selecting, projecting or joining properties for complex and structured data; (c) feature induction; (d) feature selection; and (e) mapping features from one representation to another. This implies that feature extraction is a component that should address the aforementioned issues of interaction with raw data, placing it into structure, and querying the resulting structure. Feature extraction approaches can be deterministic, such as logical query languages on relational data, or they can be information extraction tools as described before.^8^ For example, we can place all phrases, extracted from a given text based on a learned constituent parser, in a relational database and then make a deterministic query for all pairs of phrases that have a specific distance between them in the sentence.

In the NLP domain, older tools such as Fextor (Broda et al., 2013) provided an internal representation for textual data and provided a library to make queries, like asking the POS-tag of a specific word or other linguistic features relying on its fixed internal representation. Even prior to Fextor, Fex (Cumby and Roth, 2003) viewed feature extraction from a first order knowledge representation perspective. Their formalization was based on description logic where each feature extraction query was answered by logical reasoning. The commonly used machine learning/deep learning libraries provide capabilities for manipulating features as far as those are represented as vectors or matrices (thus no handling of arbitrary structures nor unstructured data) using techniques like dimensionality reduction and other vector manipulations. The recent tools specialized for learning from raw data such as natural language processing tools, of course, provide various models that can extract structures from language^9^ but different structured representations can not be easily connected to each other in a unified global structure that can be easily queried. While there has been research on each of the items (a–e) mentioned above, a unifying framework remains elusive, as does a programming environment that facilitates ML with complex and relational structures hidden in the raw data.

### 2.2. Exploiting Domain Knowledge

We use the term “knowledge” to describe the type of information that goes beyond single data items, is external to the existing data, and expresses relationships between objects and classes of objects in the real world. This is the kind of information that, for example, first order logic formalisms are able to express. Different types of domain knowledge can be distinguished based on the type of concepts, the functionality or the representation. In this article, we classify the type of knowledge based on the latter factor (the way it is expressed from the programming languages' perspective): *declarative* and *procedural* knowledge.^10^

**Declarative Knowledge**. Traditional expert systems emphasized the use of world knowledge expressed in logical form, due to its declarative nature. Although domain knowledge can convey more information than a set of data items, it is not always straightforward to account for it in classical learning approaches. In the EMR example, while the specific linguistic features of each word/phrase are part of our information about each instance, we can have some higher level knowledge over sets of phrases. For example, we know that “if an arbitrary phrase has type *person* it can not be a *location*” and that “if an arbitrary phrase is a person and another arbitrary phrase is a location, the relation between them can not be married.” Statistical relational learning models, constrained conditional models (Roth and Yih, 2004; Chang et al., 2012), and probabilistic logical languages (De Raedt et al., 2016) address this issue. Some of the current probabilistic logical frameworks are based on the classical logical reasoning using symbolic processing for recognizing the equivalence of first order logical expressions by unification algorithms and applying logical inference algorithms such as resolution. In these frameworks, for the learning part, the data items are represented coherently as grounded facts in predicate logical form. The parameters of learning models can still be trained based on the data. A typical example is Problog (De Raedt et al., 2007).

Logical representations of the domain knowledge have been used in several frameworks under the umbrella term of SRL models. These include Constrained Conditional Models (Roth and Yih, 2004; Chang et al., 2012), Bayesian Logic Programs (Milch et al., 2005; Kersting and Raedt, 2008), Markov Logic Networks (Richardson and Domingos, 2006), and Probabilistic Soft Logic models (Broecheler et al., 2010). The logical representations in these frameworks are usually grounded and generate data instances which form the underlying probabilistic graphical models of various kinds. In Roth and Yih (2004), propositional logical formulas are converted into linear inequalities that constrain the output of structured learning problems. SRL models do not necessarily consider logical reasoning. Nevertheless, the relational and logical representations provide a compact and expressive mean for higher order information that can potentially be exploited for efficient inference. Representing domain knowledge along with the data has been a major component of deductive databases such as Datalog (Gottlob et al., 1989), while expressing uncertainties in the data has been considered in probabilistic databases (Suciu et al., 2011). An example of a deductive database that represents uncertainties, is ProPPR (Wang et al., 2014), which has been augmented to learn the probabilities of the facts in the database using neural techniques in TensorLog (Cohen et al., 2017). Learning the structure of SRL models has also been considered^11^ and shown to be successful in many applications (Natarajan et al., 2014).

Logical programming is the basis of most AOP languages too—a main example is the GOLOG family (Lesp et al., 1994; Lespérance et al., 1996). The knowledge about the beliefs, capabilities and decisions of intelligent systems is declaratively programmed using such languages, and used by agent for reasoning and intelligent decision making (Shoham, 1993).

**Procedural Knowledge**. One form of procedural knowledge is the knowledge about a specific task that an intelligent agent is supposed to perform. While knowledge about the data-items, concepts and their relationships is naturally expressed via logical formalisms and in a declarative form, for some domains these representations are less convenient. For example, while the rules of a game (including the legal actions and the goal) could be described in logic, the recipe for cooking a dish or calling a person by phone are inherently procedural and include a sequence of actions. Depending on the application, we should be able to describe both types of domain knowledge in the learning models. Current programming languages take one of the two mentioned approaches, not both. For example, to program a procedure in Prolog, the code needs to be written in the form of logical rules in a way that the interpreted semantics by Prolog lead to running the intended procedure. This can make writing very simple procedures somewhat unintuitive and difficult to code properly unless the programmer is very experienced with Prolog and its formal semantics.

Using procedural knowledge in programming intelligent agents has a long history in BDI (Belief-Desire-Intention) framework (Georgeff and Lansky, 1987; Georgeff and Ingrand, 1989), which is one of the main cognitive models/architectures adopted in AOP area. A distinguishing feature of BDI and other AOPs based on BDI model (Rao and Georgeff, 1991, 1998; Rao, 1996) is to consider *plans* as the abstraction used to program agent's behavior while plans are essentially a way to specify and embed procedural knowledge about how to achieve some goal.

Using procedural knowledge representations for machine learning can have various interpretations. Sometimes, “imperative programming” refers to the way we express the training and prediction procedures. However, teaching a machine to perform a task with a sequence of steps may require one to express the procedure of the task as part of the background knowledge. The imperative task definition is different from an imperative program that hard codes the objective function of the training.

To clarify the usage of the terms in this article, even defining a task procedure subject to the learning is referred to as “declaring the procedural domain knowledge.” The procedure of a task, expressed in an imperative form, could be taken as the declaration of a specific learning model and be connected to some formal semantics with a different underlying computation from the deterministic sequential execution of a set of instructions. We also call this “declarative programming” because parts of the domain knowledge are expressed procedurally, but the execution is not deterministic and depends on the trained models. While this might be merely an issue of terminology, we believe this perspective is important to broaden the scope of declarative knowledge representation in the context of learning-based programming. Given this view, we can also call differentiable programs (Bosnjak et al., 2017) learning-based programs; however, there are severe limitations of what can be expressed in these programs. We will clarify this further when we discuss model composition in Section 2.6. An example of an imperative learning based program for the EMR task could be a basic if-then-else structure to form a pipeline of decision making. For example, if phrase *x* is a person then check phrase *y*; if phrase *y* is a location then check the relationship between *x* and *y*; and so on. This specifies a procedure for decision making although the decisions are based on learning functions. Nevertheless, it guides the formulation of a global objective function for learning.

### 2.3. High-Level Abstractions

Traditional declarative programming often considers programs as the theories of formal logic, but in general, declarative programs could be any high-level specifications of *what* needs to be done where the *how* is left to the language's implementation. All current tools and languages aim at obtaining the right level of abstraction and being declarative in that sense. We distinguish between two types of abstractions, a) *data and domain abstractions* and b) *computational and algorithmic abstractions*.

Current ML (see text footnote ^2^) and deep learning tools^12^,^13^, (see text footnote ^5^) have made a considerable progress toward being more declarative and independent from algorithms, at least for standard ML problems. Using classical ML libraries, the programmer needs to provide feature vectors and to specify only a number of parameters. The programs are written independently from the training algorithms. Retaining the high-level declarations becomes more challenging when the data becomes complex and structured as we go beyond predicting a single variable in the output. We need to use additional domain knowledge beyond data items and feature vectors.

Depending on the type of technique, various abstractions have been made based on both data and computations: (i) data and domain abstractions in terms of logical representation of the domain knowledge, (ii) data abstractions based on dependency structure of the variables, (iii) computational abstractions based on mathematical functions that form the objective of learning and inference, (iv) a combination of data and computational abstractions representing models as a procedural partial program. We describe these various perspectives and related implementations. In the following, we briefly overview the existing related work, distinguishing them by their type of abstraction.

**Logical Representation of the Domain Knowledge**. We have touched on this briefly in Section 2.2 where we described considering domain knowledge in learning. The paradigms in probabilistic logical programming and statistical relational learning use the idea of representing data and domain abstractions in terms of logical and relational representations.

**Dependency Structure of the Variables**. Probabilistic programming languages facilitate the specifications of (in)dependencies. The user declares random variables and their dependency structure and other related parameters such as distributions and density functions. The structure is specified, used declaratively, and is independent of underlying algorithms for inference and parameter estimation. The domain knowledge includes the prior assumptions about the distributions of random variables. Reconsider our EMR task. We specify the phrases as random variables after we have already obtained an appropriate representation for them. Next, we specify the dependency between each word and its label, or the labels of each word and its adjacent word. Given the data, we can then train the parameters and query probabilities of each label or do MAP inference to find the best sequence labels for the entities in a sentence. Examples of such languages^14^ are InferNet (Minka et al., 2014), Figaro (Pfeffer, 2016), AutoBayes (Fischer and Schumann, 2003), BUGS (Gilks et al., 1994), and Stan (Carpenter et al., 2017). Some of these languages are Turing-complete and support general purpose programs using probabilistic execution traces [Venture (Mansinghka et al., 2014), Angelican (Wood et al., 2014), Church (Goodman et al., 2008), and Pyro^15^]. The probabilistic logical languages provide an additional layer of abstraction on top of what probabilistic programming languages provide. They enable the user to program in terms of data and knowledge and express the dependencies at a logical and conceptual level rather than the (in)dependency structure of the random variables, which is directly used by probabilistic models. The logical representations are given semantics and interpretations that are mapped to lower level probabilistic dependency structures.

**Programming the Mathematical Objective Functions**. Typical examples of this type of abstraction are deep learning tools. The programmer does not specify the structure of the data or the dependencies between variables, but the architecture of the model based on mathematical operators, activation functions, loss functions, etc. (Abadi et al., 2016). Given the architecture of the operations, which is a computational graph in contrast to a dependency graph, the program would know how to compute the gradients and what procedure to run for training and prediction. The program specifies the objective function of the training without any concerns about taking the gradients or writing the optimization code. The declarations are connected to automated differentiation tools (Baydin et al., 2017). If we design the EMR model in this paradigm, we will need to have a vector representation of each phrase beforehand and decide how to represent the structured sentences as tensors. Deep learning tools will be able to operate on these representations and facilitate specifying the architecture of the learning models. We can specify the objective function in terms of multiplications, summations, activation functions and other diffrentiable operations. Making mathematical abstractions has been used in many other paradigms, even in probabilistic programming tools such as WOLFE (Riedel et al., 2014). Such abstractions have been used in the context of designing structured output prediction models such as SSVM (see text footnote ^3^) or with search-based inference frameworks such as Searn (Daumé et al., 2014) where the loss and predict procedures can be written in a few lines of code. In SSVM, implementing a task-specific inference algorithm is left to the programmer, while in Searn, a generic search-based algorithm for inference is proposed. The end-to-end program has a sequential and imperative structure rather than a declarative form.

### 2.4. Representing Uncertainty

Most real data is uncertain due to noise, missing information and/or inherent ambiguities. This has triggered a transition from traditional AI's logical perspective to models that support randomness and probabilities. Statistical and probabilistic learning techniques inherently address the issue of uncertainty, and this is reflected in the probabilistic programming and SRL languages (Milch et al., 2005; Richardson and Domingos, 2006; De Raedt et al., 2016). Dealing with uncertainty using probabilistic models has been added to database technology in probabilistic databases (Suciu et al., 2011) as well as some deductive databases (Wang et al., 2014; Cohen et al., 2017). It remains a challenging research question to have efficient querying capabilities while dealing with uncertainty in data.

In real-world scenarios, the uncertainty in data leads to uncertainty in executing tasks. Conventional programming languages by no means address the issue of uncertainty —a main reason why they cannot directly solve real-world problems or facilitate intelligent decision making. Uncertainty in a generic problem solving programming paradigm has been addressed in a very limited way. An example of considering uncertainty when programming for problem solving with Turing-complete capabilities can be seen in the implementations of *probabilistic logical programming* languages (Sato and Kameya, 1997; De Raedt et al., 2007; Eisner, 2008) as well as *probabilistic programming* considering randomness in the execution traces (Goodman et al., 2008; Mansinghka et al., 2014). In these frameworks, researchers have used a Turing-complete language in the background, which enables performing any arbitrary task, and have enriched it with uncertainty representation to find the best possible output when lacking evidence for finding the exact output of the program. The uncertainties are interpreted and mapped to a specific formal semantics in the existing languages. In fact, almost all current frameworks use a mapping to a specific type of probabilistic graphical models, therefore, different inference techniques based on various formalisms are often not supported.

The idea of differentiable programming can be seen as a way to deal with uncertainty in procedural programs. The issue of incompleteness is addressed by using a different type of underlying algorithm, typically that of recurrent neural networks and neural Turing machines (Graves et al., 2014). Based on this type of technique, in Bosnjak et al. (2017), for example, the sketch of an imperative program is given while the uncertain components of the program are trained given a set of input/output examples.

There is a need to address the uncertainty and incompleteness in the data and knowledge as well as in executing tasks while using various computational models and underlying algorithms.

### 2.5. Wide Range of Algorithms

The current practice of designing machine learning models for any new problems includes experimentation with a wide range of algorithms. There is no sufficient theoretical evidence to decide which learning and inference algorithms will be more effective for a specific type of application. This issue leaves the programmer with an exhaustive experimentation and trail and error. While automatic exploration is an ideal goal and the first steps have been promising, (see e.g., Thornton et al., 2013; Pfeffer et al., 2016), connecting representations to a variety of algorithms without much engineering is unexplored. Particularly, when the inputs and outputs are complex and inference over possible structures is needed, current tools do not cover various types of algorithms. The current programming frameworks mostly support a specific class of algorithms for training and inference. For example, probabilistic programming languages and SRL frameworks are based on inference and learning in probabilistic graphical models (PGM), either directed or undirected, or generic factor graphs. Probabilisitc soft logic considers a PGM too but with more scalable algorithms and more efficient solutions by forming a convex optimization problem in a continuous space for inference. LBJava, RELOOP and Saul map the inference problems under the domain's logical constraints to form integer linear programs and use efficient off-the-shelf techniques in that area to solve the inference. In LBJava and Saul, learning independent models offers the opportunity to exploit any arbitrary ML algorithm in the training phase and to perform global inference during the prediction phase. The joint training and structured learning is limited and does not cover a variety of techniques. Deep learning tools are also limited to representing differentiable objectives that are optimized based on gradient descent and back-propagation of the errors for training.

### 2.6. Model Composition

As we move toward engineering and using AI systems for increasingly complex real-world tasks, the ability to reuse, combine and chain models, and to perform flexible inference on complex models or pipelines of decision making, becomes an essential issue for learning-based programming. When designing complex models, one key question is how to compose individual models and build more complex ones based on those in the current formalisms. Reconsider our EMR task. We can design a model for classifying entities and another model for classifying the relationships. The final, global EMR model will use them as its building blocks.

The composition language can be a unified language and consistent with basic ML building block declarations. For example, we can form a global objective using the structured output prediction models and perform collective classification to solve this problem. If we have heterogeneous underlying models based on different techniques, then forming a global objective will not be straightforward as there will be multiple possibilities for combining models. This issue raises the question of whether the current tools naturally support composition or we need an additional language on top of the language for forming learning objectives. Looking back at the aforementioned frameworks, the first set of tools for classical ML do not support declarative composition. They rely on the ML and programming expertise of the users to program the model composition imperatively.

**Composition in Probabilistic Programming**. Probabilistic programming covers the aspect of composition inherently. All known and unknown variables can be declared consistently in one framework, that is, as a part of one joint probability distribution, which is factorized based on the dependency structure of the variables. The factorization of the joint probability expresses the (de)composition semantics for learning and inference. Thus, the way that we compose complex models is limited to expressing more global dependencies, and the same dependency structure is used for both training and prediction. However, this is not always sufficiently expressive for building complex models and pipelines of decision making. For example, we can not compose arbitrary parts based on verifying the validity of certain conditions.

**Composition in CCMs**. When designing constrained conditional models (CCMs) in languages such as Saul or LBJava, we need to program the two components of local learning declarations and global constraints specifications. The composition can be done consistently as far as it can be formulated by imposing global constraints and building global models. The current implementations based on CCMs (Rizzolo and Roth, 2010; Kordjamshidi et al., 2015) can model pipelines and model composition by considering the learning models as first class objects where their outputs can be used to form new learning models and new layers of abstractions. Although, in the frameworks that are designed as libraries of general-purpose languages, the compositions can be made by the programmer, a composition language with well-defined semantics is missing and will provide a better way to design complex models with explicit structures, end-to-end. In other words, with CCM-based frameworks a well-defined composition language is still missing.

**Composition in Deep Neural Models**. The deep learning tools rely on general purpose programming environments and the ML and programming skills of users to compose models imperatively. They provide a way to design single models, though CapsNets (Sabour et al., 2017) made a first step toward learning compositions in deep networks. Designing Neural Module Networks is another related direction to build neural modules per domain concepts and compose them explicitly and dynamically for language and vision understanding (Andreas et al., 2016). These models, though modular and composable, still rely on end-to-end neural training based on continuous representations.

**Programs Seen as Compositions of Models**. While the composition of the trained models is helpful in designing and programming complex models, one new issue arises. Can we parameterize the programs that include learning-based components and in turn learn the composition itself? This is a less established line of research. It is not clear how the structure of the program can be represented or what the parameters of the program will be. Differentiable programs could be seen as an important step in this direction. There are very recent developments in the area of image processing and physical simulation that are following this direction of imperative and differentiable programming for designing end-to-end learning models (Li et al., 2018; Hu et al., 2020). The program includes the pipeline of parametrized operations that can be trained with data. This research relates to program synthesis in the sense that we learn a program from inputs and outputs. From a different angle, it can be seen as learning the parameters of the composition of learning-based components where we can provide the structure or the schema of the program and learn parts of it. The latter perspective should be distinguished from program synthesis because the target programs that we learn do not necessarily perform tasks with deterministic nature such as sorting. The programs can only estimate an output given a partial structure and make inference. The intelligent (learning-based) programs are unlikely to be fully determined with a fixed structure.

## 3. Declarative Learning-Based Programming: An Integration Paradigm

The conclusion of this survey will not be to promote any of the existing frameworks but to advocate for an integration paradigm. As pointed before, we use the term *(Declarative) Learning Based Programming* only to refer to such an ideal paradigm. While existing frameworks do address some of the capabilities (1)-(6) described in Section 1.1, there is still a need to integrate these aspects in unified frameworks to design AI systems.

**Figure 2:**
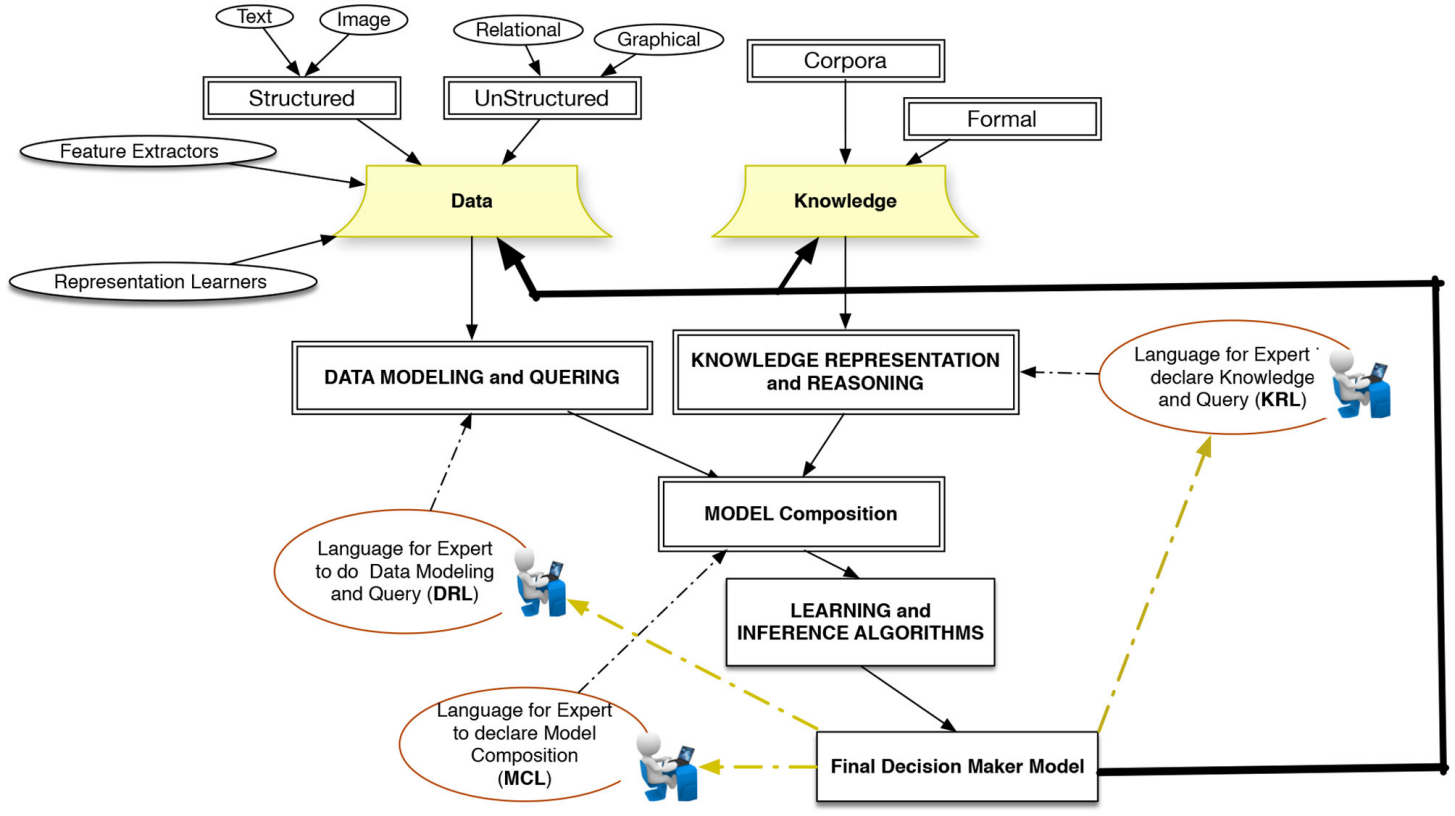
The main components and sub-languages of a learning-based programming system.

We argue here for a paradigm in which *learning from data* is the central concept and extends the capabilities of designing intelligent systems around this concept. Such a paradigm, should address the above-mentioned challenges accordingly and allow programming to construct complex configurations using basic learning building blocks. Remaining as a survey article, we avoid proposing a detailed architecture for supporting such an ideal system, however, Figure 2 shows a rough sketch of a platform that can address the application requirements from an AI-systems development perspective. The platform integrates the capabilities for working with heterogeneous data and knowledge from various resources. This implies that there will be a need for a data modeling and representation language (DLR), a knowledge representation language (KRL), and a model composition language (MCL). These three languages should have access to a set of learning and inference algorithms and allow domain experts to design models interactively. The output of the intelligent models is either new data or new knowledge that is added back into an evolving intelligent system. In the previous sections, we reviewed the current frameworks and the type of abstractions that they provide. To conclude, here we argue for the need to new abstractions with the following characteristics to further facilitate programming and interaction with AI-systems.

**Abstractions That Are Independent From Computations**. Learning-based programming (Roth, 2005), requires data and programmatic abstractions, hiding the algorithmic details and even hiding high-level algorithmic abstractions. Learning is a mapping from one data abstraction layer to another given the data instances, starting from raw data. The user needs to specify the intended abstractions for an application in hand, and the system should figure out how to perform the actual mappings. While this abstraction follows the similar ideas in logical formalisms, here we are not limited to logical predicates. The primitives can be concept-learners that are represented by arbitrary functions. The mapping computations are not limited to logical reasoning mechanisms, and heterogeneous learners can take the data and learn the mappings. LBJava (Rizzolo, 2011b) was the first attempt to implement this idea, based on the CCM (Chang et al., 2012) computational model. Learners are first class objects, and the domain knowledge also represented in terms of data abstractions and can be used by learners to make global and consistent mappings. RELOOP (Kersting et al., 2017) took a similar approach from a mathematical programming perspective, combining relational and mathematical programming aspects embedded within an imperative language (in this case, Python). Saul (Kordjamshidi et al., 2015) has been proposed with a similar computational model and the possibility of joint training of global models. Saul is in the form of a library without the data-driven compilation step, and it comes with explicit support for the representation of the data as a graph for relational feature engineering. The data graph representation helps to specify domain concepts and their relationships. Some concepts are connected to sensors abstracting away from raw data and some are concept learners. The *DomiKnowS* neuro-symbolic framework proposed recently (Faghihi et al., 2021) and follows Saul ideas in which the modeling starts with domain specification in terms of concepts and relationships independent from the underlying computations.

**Abstractions That Facilitate Algorithmic Coverage**. Most of the frameworks mentioned in the previous sections have limited coverage of supported class of algorithms. While some of these are more flexible than others in supporting heterogeneous computational building blocks, training complex configurations with structured learning is addressed with one class of techniques in each framework—for example, either of probabilistic inference, integer linear programming, or dynamic programming and search. Note that classical machine learning tools that perform classification/regression/clustering based on vector representations of data do not suffer from the algorithmic coverage. The coverage issue arises when we need to support inference based on a specific representation language. This will limit the semantics of each formalism and the type of algorithms that can be used. To deal with this issue, the learning model abstractions should be based on the data abstractions, domain knowledge representation and generic problem specification. This level of representation will be independent from learning and inference algorithms and can be connected to various computational models. In contrast, the representations based on computational abstractions (such as deep learning tools) are more bounded to the type of underlying techniques for computations and impose more limitations on the algorithmic coverage. In the current tools, all optimizations are based on gradients and the computational building blocks are neural networks modules. If we need to perform gradient-based training along with probabilistic inference, no generic framework and representation language supports both class of techniques/algorithm.

**Abstractions That Help in Closing the Loop of Data to Knowledge**. Intelligent systems need to evolve over time. As they receive more data and knowledge, they find better abstractions of the data, as illustrated by NELL (Mitchell et al., 2015), Never-Ending Language Learning. Representations of the learning models based on the data and knowledge will naturally support feeding the current models (which will be trained concepts) to obtain new abstraction layers. Since each concept is related to a learning model (i.e., a concept learner), combining concepts to form new concepts will be equivalent to composition of learning models to create new learning models. This is a natural way to support model composition. In other words, there will be a direct connection between how we compose models and how we compose real-world concepts. Such abstractions will help to close the loop of moving from data to knowledge and exploiting knowledge to generate new concepts. How to implement such an idea, will be a research question for an ideal learning based programming framework.

**Abstractions That Help With Learning the Programs**. While the goal of ML is to write programs that can learn to do a task or make a decision, a more ambitious goal would be to learn the structure of the programs from the data. From the classical ML perspective, this relates to structure learning. An example is learning the dependency structure of the probabilistic models such as Bayesian networks, see Koller and Friedman (2009). Another dimension of the problem is learning features or feature induction, which has been investigated in the classical machine learning community for years (Molina et al., 2002). Learning global constraints by analyzing the data is another line of work leading toward learning the structure of the learning models (Bessiere et al., 2017), which is related to traditional rule learning models that can guide the issues of learning the programs.

In the programming languages community, this problem is closely related to program synthesis from the inputs and outputs. From the classical AI perspective, this is also related to inductive logic programming and program induction (see e.g., Muggleton and De Raedt, 1994). These ideas go beyond learning propositionalized rules and are about learning logical programs. They can be seen as a set of rules augmented by global formal semantics for symbolic processing of logical expressions (also of high order) to evaluate their syntactic and semantic equivalence to be able to unify them (i.e., unification), and perform logical reasoning to entail more knowledge. In fact, inductive logic programming can be considered to be at the same level as other learning algorithms where the structure of the model (i.e., the program) is already given. This structure is usually in the form of a language bias, which is very different from the way the model's structure is defined in (non-relational) statistical learning paradigms. The ultimate case of program induction, learning the programs in the framework of logic, is when the domain predicates are not fully specified but need to be invented during learning (Stahl, 1995). A logical programming language or a classical general purpose programming language, even if it is Turing equivalent, will not be able to solve an AI-complete^16^ problem. Even if the structure of a learning model is found, the parameter learning is an additional challenge in this context to address incompleteness and uncertainty for solving problems intelligently. This is the major difference between the work done in the scope of program synthesis compared to learning programs that are intended for AI research.^17^

Our choice of program content and representation, discussed in Section 2.3, is a key factor that influences the way we approach learning the programs themselves and the types of techniques that will be developed in this direction. Depending on the representation of the programs, learning programs can involve learning deep architectures, learning dependence structures or learning classic machine learning features.

**Other Issues From AI and Learning-Based Systems Perspective**. The present aticle focuses on the issues related to appropriate and easy-to-use abstractions and coverage of various formalisms for learning-based programming. It does not investigate many other requirements and issues for the platforms that eventually employ these declarative languages for designing AI systems (Stoica et al., 2017). At least, we need to solve similar problems that we face for example in database management systems when designing AI systems. It is imperative to have learning-based management systems that can deal with security and privacy of data as well as learning models, scalability of learning and inference, distributed and parallel implementations, concurrency and more. There are new issues such as fairness and explainability to be addressed in AI and learning-based management systems. Generating the supervision signals is another important challenge; there is a need to constantly collect weak and incidental signals independently of specific tasks and relate them on the fly to solve a task without supervision (Roth, 2017). Moreover, though we argue for a declarative programming paradigm as an interface to interact and design the AI systems, a higher level and more ambitious interface will be natural interaction (Gluck et al., 2018). Natural interactions such as speech, language and visual demonstrations can be used as a media to transfer data and knowledge to models and develop life-long learning intelligent systems.

## 4. Conclusion

Triggered by the emerging research area of Systems AI—the computational and mathematical modeling of complex AI systems—we provided an overview on *declarative learning-based programming* languages as a central component of such a mission and as an interface to interact with AI systems for designing, training and using them for real-world decision-making and task performance. We discussed the related works that can help to design such a language covering (a) the type of abstraction that they make over the data and computations, (b) the type of techniques that they cover for learning and reasoning/inference (c) the way they address the interaction with data and the issue of incompleteness and uncertainty (d) the way that those facilitate designing complex models by composition of simpler models. More importantly, we reviewed the missing components of the existing models, and the necessity of collaborations to develop an integrated framework for Systems AI. Finally, we emphasized that working on the declarative programming languages that describe the programs in terms of data, knowledge and declaring task procedures will pave the way for training AI systems by natural interactions (Gluck et al., 2018). The declarative programs can be seen as intermediate representations that intelligent systems can receive directly from the programmers, or ideally learn/infer them from natural interactions in the real world.

## Data Availability Statement

The original contributions presented in the study are included in the article/supplementary files, further inquiries can be directed to the corresponding authors.

## Author Contributions

PK prepared the first draft of this survey by reviewing the literature. She continued discussion and communication of the work with DR and KK. The ideas related to learning based programming are related to the outcome of the research by DR, PK, and KK on designing Declarative Learning platforms. DR has reviewed the manuscript evaluated, added, modified the content, participated in discussions about the content of the article, and contributed to editing the write up. KK has revised the first draft, evaluated, and added content to it. He participated in discussions about the content of the article and contributed to editing the write up. All authors contributed to the article and approved the submitted version.

## Funding

This project is partially funded by the Office of Naval Research (ONR) grant #N00014-20-1-2005.

## Author Disclaimer

Any opinions, findings, and conclusions or recommendations expressed in this material are those of the authors and do not necessarily reflect the views of Office of Naval Research.

## Conflict of Interest

The authors declare that the research was conducted in the absence of any commercial or financial relationships that could be construed as a potential conflict of interest.

## Publisher's Note

All claims expressed in this article are solely those of the authors and do not necessarily represent those of their affiliated organizations, or those of the publisher, the editors and the reviewers. Any product that may be evaluated in this article, or claim that may be made by its manufacturer, is not guaranteed or endorsed by the publisher.

## Footnotes

^1^Though Lisp was not originally designed for logic programming, in later versions McCarthy decided to enrich it for AI programming.

^2^ http://scikit-learn.org/stable/

^3^ www.cs.cornell.edu/people/tj/svm_light/svm_struct.html

^4^ http://cogcomp.org/page/software_view/JLIS

^5^ https://pytorch.org/

^6^Sentence splitting itself is a non-trivial task that may require learning, but in our example, we assume that sentences are given.

^7^See Kordjamshidi et al. (2015) and Rizzolo and Roth (2010) for sensor definition.

^8^"Deterministic" is not the exact technical term here, but related to the notion of deterministic queries that retrieve a fixed stored value rather than returning a value that is computed by a function and can change later; a non-deterministic query in our case can be a query calling a trained machine learning model.

^9^https://www.nltk.org/, https://allenai.org/allennlp

^10^While we use the term imperative programming in contrast to declarative programming, we refer to the related type of knowledge as procedural knowledge as it is usually used in other AI communities, particularly, in cognitive architectures.

^11^ https://starling.utdallas.edu/software/boostsrl/wiki/

^12^ https://www.tensorflow.org

^13^ openai.com

^14^ probabilistic-programming.org

^15^ pyro.ai

^16^ https://en.wikipedia.org/wiki/AI-complete

^17^While classical program synthesis is a complex task itself and might be solved with machine learning and AI techniques, this is not the subject matter of the discussion here. We point to this topic because synthesizing the learning based programs can be related to learning the structure of the machine learning models.
